# Supplementary material for: Polo-like Kinase 1 Predicts Lymph Node Metastasis in Middle Eastern Colorectal Cancer Patients; Its Inhibition Reverses 5-Fu Resistance in Colorectal Cancer Cells
Source: Cells. 2024 Oct 14;13(20):1700. doi: 10.3390/cells13201700 (PMC11506015; doi:10.3390/cells13201700)
Supplement: Supplementary file 1 [file cells-13-01700-s001.zip › cells-3215824-supplementary.pdf]

Figure S1

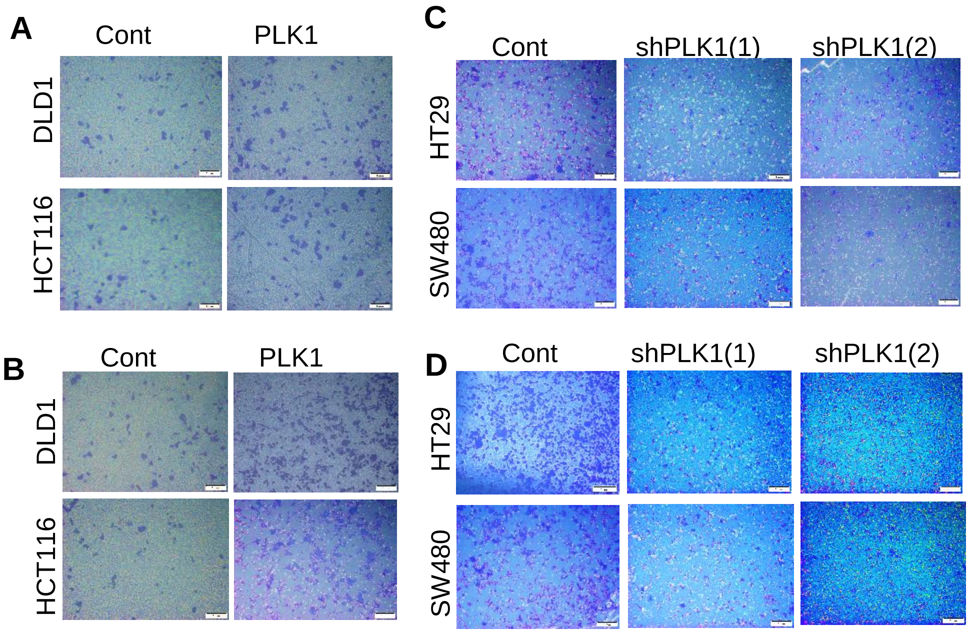

Supplementary Figure 1. PLK1 inhibition decreases metastatic potential of CRC cells. (A) Overexpression of PLK1 enhances the invasive ability of CRC cells. **(B)** Overexpression of PLK1 increases the migratory capacity of CRC cells. **(C)** Silencing of PLK1 decreases invasion. **(D)** Silencing of PLK1 decreases migration.

Figure S2

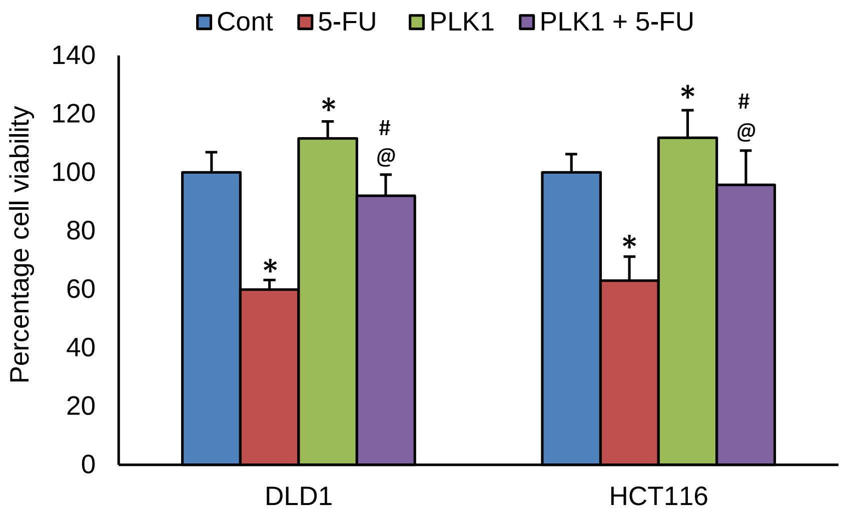

Supplementary Figure 2. Forced expression of PLK1 induces chemoresistance in CRC cells. PLK1 overexpressing cells were treated with or without 5-Fu for 48 hours, and cell viability was determined using the MTT assay (n=6). *Significant compared to control, @ Significant compared to 5-Fu alone, # Significant compared to PLK1 alone.
